# The Effect of Low-Fat and Low-Carbohydrate Diets on Weight Loss and Lipid Levels: A Systematic Review and Meta-Analysis

**DOI:** 10.3390/nu12123774

**Published:** 2020-12-09

**Authors:** Shreya Chawla, Fernanda Tessarolo Silva, Sofia Amaral Medeiros, Rania A. Mekary, Dina Radenkovic

**Affiliations:** 1 Faculty of Life Sciences and Medicine, King’s College London, London WC2R 2LS, UK; shreya.chawla@kcl.ac.uk; 2 Faculdade de Medicina da Universidade de São Paulo, São Paulo 01246-903, BR, Brazil; fernanda.tessarolo@fm.usp.br (F.T.S.); sofia.amaral@fm.usp.br (S.A.M.); 3 School of Pharmacy, MCPHS University, Boston, MA 02120, USA; RMekary@hsph.harvard.edu; 4 Nutrition Department, Harvard TH Chan School of Public Health, Boston, MA 02120, USA; 5 HOOKE London, 11-15 Farm Street, London W1J 5RG, UK

**Keywords:** low carbohydrate diet, low fat diet, cardiovascular outcomes, weight loss, lipid panel, cholesterol, preventative medicine, cardiology, nutrition

## Abstract

Background: The rise in obesity has emphasised a focus on lifestyle and dietary habits. We aimed to address the debate between low-carbohydrate and low-fat diets and compare their effects on body weight, low-density lipoprotein cholesterol (LDL), high-density lipoprotein cholesterol (HDL), total cholesterol, and triglycerides in an adult population. Method: Medline and Web of Science were searched for randomised controlled trials (RCTs) comparing low-fat and low-carbohydrate diets up to September 2019. Three independent reviewers extracted data. Risk of bias was assessed using the Cochrane tool. The meta-analysis was stratified by follow-up time using the random-effects models. Results: This meta-analysis of 38 studies assessed a total of 6499 adults. At 6–12 months, pooled analyses of mean differences of low-carbohydrate vs. low-fat diets favoured the low-carbohydrate diet for average weight change (mean difference −1.30 kg; 95% CI −2.02 to −0.57), HDL (0.05 mmol/L; 95% CI 0.03 to 0.08), and triglycerides (TG) (−0.10 mmol/L; −0.16 to −0.04), and favoured the low-fat diet for LDL (0.07 mmol/L; 95% CI 0.02 to 0.12) and total cholesterol (0.10 mmol/L; 95% CI 0.02 to 0.18). Conclusion and Relevance: This meta-analysis suggests that low-carbohydrate diets are effective at improving weight loss, HDL and TG lipid profiles. However, this must be balanced with potential consequences of raised LDL and total cholesterol in the long-term.

## 1. Introduction

Being overweight is associated with major long-term conditions including diabetes, cardiovascular disease (CVD), and several types of cancers, implicating the need to address obesity as a major risk factor for the most common chronic conditions [1,2,3]. The burden of obesity has escalated dramatically in recent years; the World Health Organisation (WHO) reported that obesity nearly tripled between 1975 and 2016 [4]. The growing prevalence of obesity, combined with diabetes estimates rising by 51% by 2045 [5] emphasise the need to undertake urgent action to reduce the burden of the obesity pandemic and the consequent rise in cardiovascular and associated diseases.

Lifestyle modification and dietary change can simultaneously alter several risk factors and thus reduce the risk of cardiovascular disease [6]. Despite great emphasis on the impact of a “healthy diet”, there remains fervent debate on which diets best address obesity and cardiovascular health [7]. The American Heart Association recommends a diet emphasising the intake of vegetables, fruits, legumes, nuts, whole grains, and fish to reduce cardiovascular risks [8]. The National Institute for Health and Care Excellence (NICE) have similar guidelines and have suggested a shift towards a Mediterranean-style diet to prevent obesity and cardiovascular risk [9,10]. However, these recommendations are often ambiguous as the exact compositions of these diets are not well-defined. Moreover, in recent years there has been a rise in popularity of carbohydrate restriction diets (Atkins, Eddies, Zone, South Beach) [11]. This is in stark contrast to the low-fat dietary guidelines which were implemented in the US in the 1970s [12] and remained as clinical practice recommendations until recently [13]. The low-fat dietary guidelines also coincided with the beginning of the obesity pandemic with a significant rise in the national obesity rate (body mass index ≥ 30) amongst adults from 14.5% in 1971–1974 to 35.3% in 2011–2012 [14]. Although no causal relationship has been determined, it is important to understand trends between recommendations and obesity rates to strengthen future dietary guidelines. The growing burden of disease and unclear recommendations have allowed the weight loss industry to become increasingly lucrative and fostered claims of certain diets being superior without sufficient evidence [15]. 

Overall, we observe that the public is provided with evolving and often broad dietary recommendations. Moreover, as low-carbohydrate diets are increasing in popularity among the public as potentially more efficient means of weight loss, there is a need to study these diets and inform both the clinicians and the general public about the systemic effects of such dietary changes on weight and markers of cardiovascular risk such as lipid profile. Therefore, the aim of this meta-analysis was to provide class A recommendations on the effect of macronutrient composition of diets on cardiovascular risk factors. We aimed to examine the impact of low-carbohydrate vs. low-fat diets on weight change and lipid panels including low-density lipoprotein cholesterol (LDL), high-density lipoprotein cholesterol (HDL), total cholesterol, and triglycerides in an adult population.

## 2. Materials and Methods 

The systematic review was reported in accordance with the 2009 PRISMA Statement and all steps of the PRISMA checklist were completed. The review protocol was registered with PROSPERO in February 2019 (registration number CRD42019123319). 

### 2.1. Search Strategy

MEDLINE (PubMed) and all databases across the Web of Science were searched up to September 2019 using search terms related to low-carbohydrate and low-fat diets and related cardiovascular outcomes (Supplementary File 1).

The aim of our search was to identify randomised clinical trials analysing the effect of low-carbohydrate vs. low-fat diets on body weight and other cardiovascular risk factors. The search was limited to English-language and to adult human randomised controlled trials published up until September 2019. Two independent authors (S.C., R.A.) screened the titles and abstracts of articles against the inclusion and exclusion criteria. Subsequently, full texts were reviewed against eligibility criteria for final selection. Any disagreements between the two authors were resolved by discussion or by consulting a third author (D.R. or S.M.).

### 2.2. Study Selection, Inclusion and Exclusion Criteria

Studies selected included randomised controlled trials in adults examining and comparing low-carbohydrate to low-fat diets. The studies included details of macronutrient composition and outcomes including weight loss as a primary outcome. Additionally, variations in other cardiovascular risk factors such as high-density lipoprotein cholesterol (HDL), low-density lipoprotein cholesterol (LDL), total cholesterol (TC), and triglycerides (TG) were included. As per our inclusion criteria, low-carbohydrate diets were defined to have <40% carbohydrate content and low-fat diets were defined to have <30% total fat content. These limits were chosen upon consulting the literature regarding low-carbohydrate [16], and low-fat diets [17,18,19], and consensus was reached between the authors. 

Studies were excluded if they were conducted in children or adolescents (<18 years) as well as in populations with significant comorbidities such as diabetes, cancer, chronic obstructive pulmonary disease, or other cardiovascular diseases. Studies were also excluded if they were not randomised controlled trials. Studies that did not include an intervention arm with ≤40% carbohydrates and another arm with ≤30% fat were excluded in order to comply with our inclusion criteria. Furthermore, studies examining glycaemic index (GI) specifically without modification of macronutrient composition were excluded as GI has not clearly been determined to be a reliable modifier of cardiovascular health [20].

### 2.3. Data Extraction 

A pre-designed excel sheet was used to extract and organise the data into categories by 3 independent authors (S.C., S.M., and F.T.). These included (1) number of participants (2) participant and intervention details, i.e., age, body mass index (BMI), follow-up period (3) energy consumption including macronutrient composition, presence of calorie restriction, prescription of physical activities, (4) outcome measures including weight loss, LDL, HDL, total cholesterol, triglycerides (5) risk of bias and study limitations. If data were not available in numerical format, the relevant authors were contacted for further information. The three crossover trials only provided overall results for low-carbohydrate and low-fat, thus these were extracted. 

### 2.4. Risk of Bias Assessment

Four authors (S.C., R.A., F.T., and S.M.) independently assessed the risk of bias of each included trial using the Cochrane tool for assessing risk of bias [21]. Any disagreements were discussed among the authors and if consensus was not reached, another senior author (D.R.) gave a final judgement. The domains of the tool included “randomisation process”, “deviations from intended interventions”, “missing outcome data”, “measurement of the outcome”, “selection of the reported result”. Each domain was assessed to have either low risk, high risk, or some concerns. Overall risk of bias was classified as low if there was low risk of bias for all domains, unclear if there was low or unclear risk of bias for all key domains, and as high if there was high risk of bias for one or more key domains according to the suggestions by the Cochrane tool for assessing the risk of bias [21].

Systematic adherence to a pre-defined protocol for study search, screening, data extraction, and analysis was carefully implemented to minimize bias. Potential publication bias was identified using a funnel plot for visual determination of asymmetry, as well as Begg’s [22] and Egger’s [23] tests for statistical significance. When publication bias was indicated, the trim-and-fill method was used to impute the potentially missing studies and recalculate the imputed pooled effect estimate, while acknowledging the limitation of such a method that it assumes the source of asymmetry is due to publication bias per se and not to other reasons.

### 2.5. Data Analysis 

Across the trials, results for weight loss were expressed in kilograms and results for BMI were in kg/m^2^. Furthermore, we converted means for total daily energy intake to kilocalories per day. Additionally, LDL and HDL were expressed in mmol/L or mg/dL; where required, we converted mg/dL into mmol/L. Where the confidence interval (CI) or standard error (SE) were given for the means, standard deviation was calculated. Where the standard deviation and mean were given as % change, the standard deviations were imputed from the remaining studies. Imputation was necessary for four studies [24,25,26,27].

In every original study, we calculated or extracted the average body weight change from baseline for the low-carb arm and the low-fat arm to derive the average difference between the 2 arms, comparing low-carbohydrate to low-fat arm. The pooled weighted mean difference and its 95% confidence interval was then calculated in a meta-analysis using the DerSimonian and Laird random-effects model [28] and stratified by follow-up period (1–3 months; 3–6 months; 6–12 months and >12 months). Similar methods were adopted for each of the LDL, HDL, total cholesterol, and TG outcomes. The analysis was performed in Comprehensive Meta-Analysis version 3.

## 3. Results

**Figure 1:**
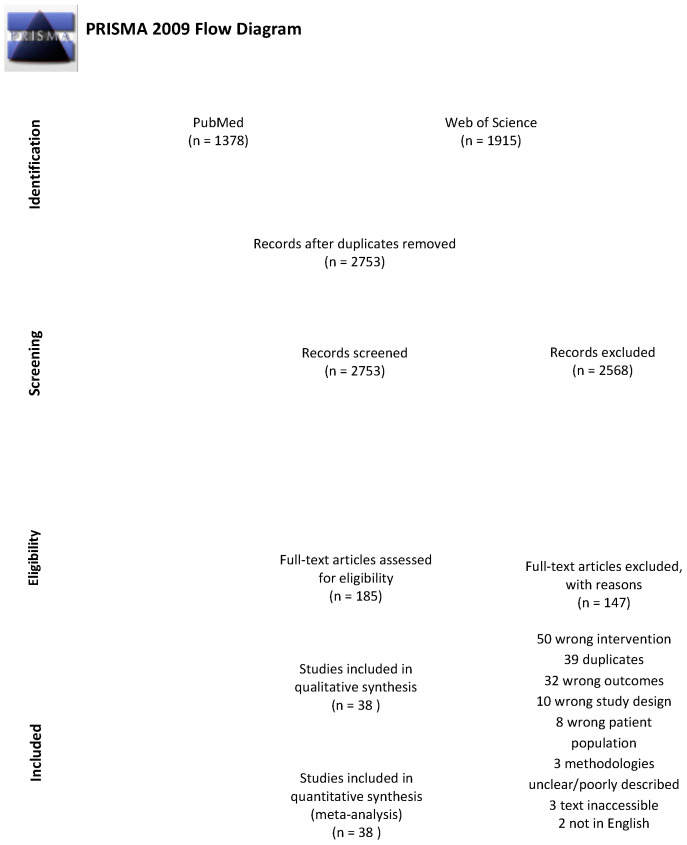
Preferred Reporting Items for Systematic Reviews and Meta-Analyses (PRISMA) flow diagram of included articles.

The search strategy resulted in 2753 articles once duplicates were removed. Of these, 2568 were excluded in title and abstract screening as they did not meet the selection criteria. Thus, 185 articles were retrieved for full text reviews; of these 147 were excluded. Therefore, 38 trials were identified for inclusion in our review (Figure 1) [7,11,24,25,26,27,29,30,31,32,33,34,35,36,37,38,39,40,41,42,43,44,45,46,47,48,49,50,51,52,53,54,55,56,57,58,59,60].

### 3.1. Characteristics of Included Trials

Characteristics of all studies are summarised in Table 1. All studies were parallel-group or crossover RCTs. Due to the nature of dietary intervention studies, none of the studies were blinded. Study duration ranged between 1–24 months and our primary meta-analysis included 6499 adults. Mean age of the participants varied from 33 to 58 years and mean BMI varied between 22 and 43.6 kg/m^2^ (Table 2). The intensity of diets varied from providing nutritional information about diets prescribed to intensive one-on-one counselling with a dietician and food provision. Caloric restriction was a component of 19 studies (Table 2) and food was provided (for a portion of, or for the complete duration) in six studies [25,34,35,42,54,58]. Participants were prescribed physical activity ranging from general exercise recommendation to prescribed exercise training programs (Table 2).

### 3.2. Weight Loss (kg)

**Figure 2:**
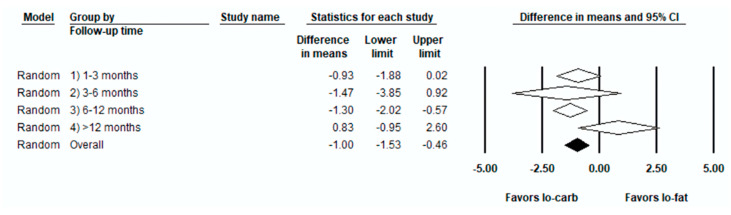
Forest plots showing weighted mean differences (WMD) and 95% CI across all studies and time periods for weight loss.

The random-effect meta-analysis of the results (shown in Figure 2) revealed an average difference in weight loss favouring participants who ate a low-carbohydrate diet overall (−1.00 kg; 95% CI −1.53 to −0.46; I^2^: 88.8%; 59 studies). When analysing by time category, the weighted mean difference (WMD) favoured the low-carbohydrate at 6–12 months (mean difference −1.30 kg; 95% CI −2.02 to −0.57; I^2^: 57.4%; 17 studies); however, there was no apparent statistically significant difference in weight loss between low-carb and low-fat diets at 1–3 months (−0.93; 95% CI −1.88 to 0.02; I^2^: 84.5%; 27 studies), 3–6 months (−1.47; 95% CI −3.85 to 0.92; I^2^: 96.1%; 13 studies), and beyond 12 months (0.83; 95% CI −0.95 to 2.60; I^2^: 0%; two studies); nevertheless, the *P*-interaction—comparing the four time categories—was not statistically significant: 0.18. 

### 3.3. Lipids

**Figure 3:**
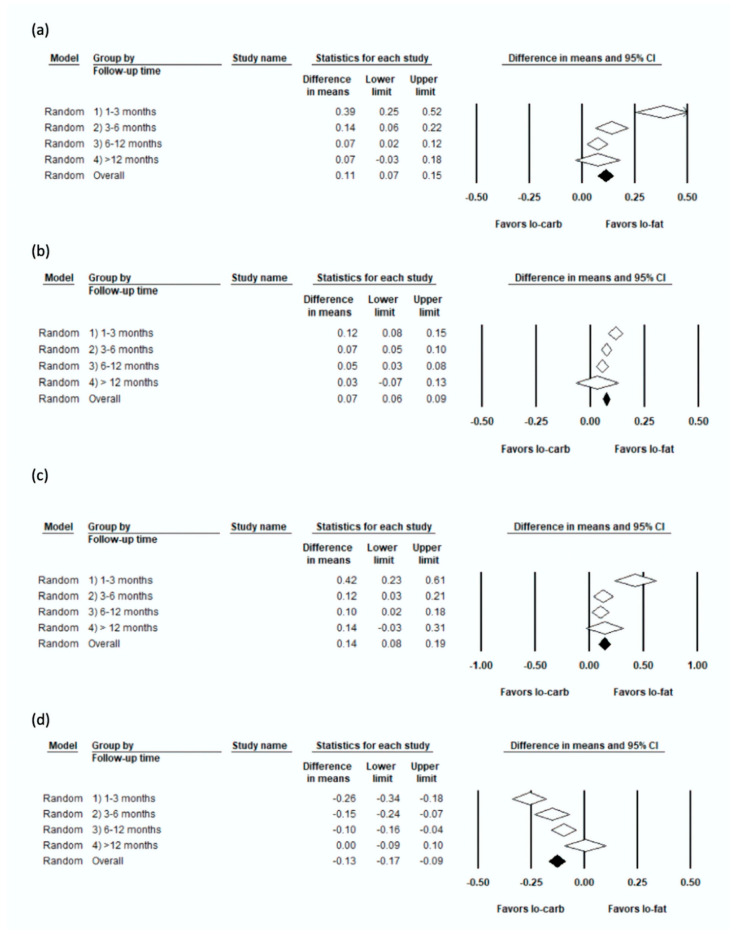
Forest plots showing weighted mean differences (WMD) and 95% CI across all studies and time periods for (**a**) Low-density lipoprotein cholesterol (LDL) cholesterol, (**b**) High-density lipoprotein cholesterol (HDL) cholesterol, (**c**) Total cholesterol, (**d**) Triglycerides.

The results of the weighted-mean-difference for LDL, HDL, total cholesterol, and triglycerides are shown in Figure 3. 

### 3.4. LDL (mmol/L)

The pooled WMD in favour of low-fat was statistically significant at 1–3 months (0.39; 95% CI 0.25 to 0.52; I^2^: 82.7%; 28 studies), 3–6 months (0.14; 95% CI 0.06 to 0.22; I^2^: 59.1%; 22 studies), and 6–12 months (0.07; 95% CI 0.02 to 0.12; I^2^: 24.7%; 18 studies); however, results were not statistically significant beyond 12 months (0.073; 95% CI −0.032 to 0.178; I^2^: 0%; two studies); the *P*-interaction comparing the four time categories was statistically significant <0.01.

### 3.5. HDL (mmol/L)

Findings in favour of low-carbohydrate were statistically significant at 1–3 months (0.12; 95% CI 0.08 to 0.15; I^2^: 67.2%; 28 studies), 3–6 months (0.07; 95% CI 0.05 to 0.10; I^2^: 56.4%; 22 studies), 6–12 months (0.05; 95% CI 0.03 to 0.08; I^2^: 68.4%; 18 studies), but not beyond 12 months (0.03; 95% CI −0.07 to 0.13; I^2^: 81.5%; 2 studies); *P*-interaction comparing the four time categories: 0.03.

### 3.6. TC (mmol/L)

The analysis of TC yielded similar results to LDL. The WMD favoured low-fat significantly at 1–3 months (0.42; 95% CI 0.23 to 0.61; I^2^: 84.4%; 23 studies), 3–6 months (0.12; 95% CI 0.03 to 0.21; I^2^: 57.5%; 17 studies), and 6–12 months (0.1; 95% CI 0.02 to 0.18; I^2^: 49.0%; 14 studies), but not in the >12 months category (0.14; 95% CI −0.03 to 0.31; I^2^: 0%; one study); *P*-interaction comparing the four time categories: 0.02.

### 3.7. TG (mmol/L)

The analysis of TG yielded similar results to HDL. The WMD favoured low-carbohydrate significantly at 1–3 months (−0.26; 95% CI −0.34 to −0.18; 28 studies), 3–6 months (−0.15; 95% CI −0.23 to −0.07; 22 studies), 6–12 months (−0.10; −0.16 to −0.04; 16 studies); however, results were not statistically significant in the >12 months category (0.004; 95% CI −0.09 to 0.10); *P*-interaction comparing the four time categories <0.01.

### 3.8. Risk of Bias and Publication Bias

**Figure 4:**
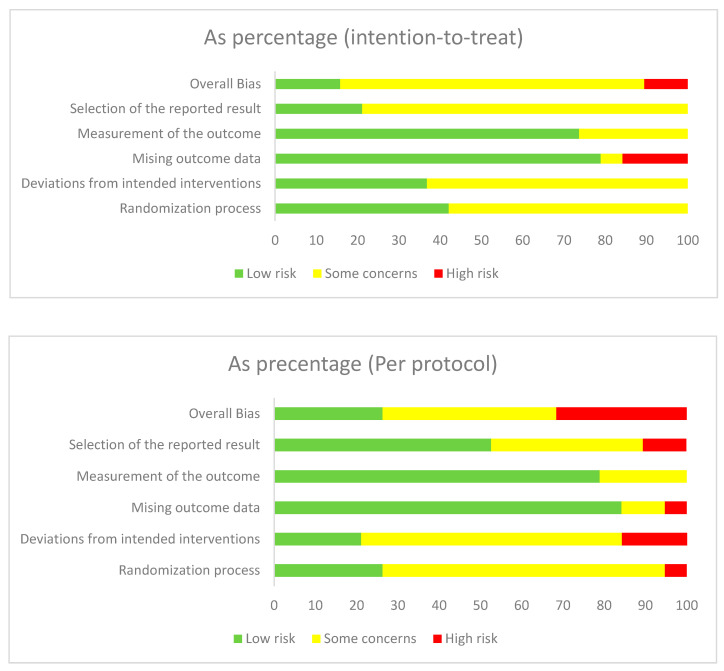
Quality assessment performed by authors on each risk of bias item presented as percentage across all included studies.

Full results from the risk of bias assessment are provided in Supplementary File 2. Most studies did not report on methods of allocation concealment and a risk was posed due to the inability to blind participants and staff due to the nature of nutritional studies. A summary of the proportion of trials in low, unclear, and high bias subdivided by intention-to-treat and per protocol studies is shown in Figure 4. 

Publication bias was examined visually through funnel plots by plotting standard error against difference of mean. While the funnel plot looked slightly asymmetrical for weight, LDL and HDL, it looked very asymmetrical for total cholesterol and triglycerides (Supplementary File 3). Using Begg’s test, possible publication bias was detected for LDL, total cholesterol, and triglycerides (all *p* < 0.01). Egger’s linear regression test also suggested possible publication bias for weight (*p* = 0.04), LDL (*p* < 0.01), HDL (*p* = 0.03), total cholesterol (*p* = 0.01), and TG (*p* < 0.01). The trim and fill method was attempted for each of the outcomes using the random-effect model, and only total cholesterol and HDL were imputed with potentially missing studies; six studies were imputed for total cholesterol and 18 for triglycerides, each to the right of the pooled mean difference. Although the imputed mean difference slightly increased for total cholesterol (0.25 (0.18, 0.33) vs. 0.20 (0.13, 0.27)) and TG (−0.10 (−0.15, −0.05) vs. −0.18 (−0.22, −0.13)) in a way towards favouring low-fat diet, the direction of the imputed results was not different from the original results. 

## 4. Discussion

In this meta-analysis of RCTs, we compared the effects of low-carbohydrate vs. low-fat diets on weight loss and cardiovascular risk factors including LDL, HDL, total cholesterol, and triglycerides. In comparison to the low-fat group, the participants on low-carbohydrate diets experienced a statistically significant greater reduction in body weight and triglycerides, and a greater increase in HDL overall. Results at 24 months were not significant for any of the variables, although the small number of studies might have contributed to this. Participants on low-carbohydrate diets experienced a greater significant increase in LDL-cholesterol and total cholesterol overall with results not being significant beyond 12 months, potentially due to lack of power.

Our findings suggested that low-carbohydrate diets were more beneficial than low-fat diets for weight loss, HDL, and triglycerides in the short-term. Nevertheless, this benefit must be balanced with potential harms from high-fat diets causing dyslipidaemia in the form of raised LDL and total cholesterol in the long-term. Thus, choice of diet should be tailored according to the patient’s baseline levels. 

The findings of our meta-analysis did not support the former view that only the high-fat diet carried negative health consequences. [13,61] Our findings were in line with meta-analyses conducted by Mansoor et al. [62] (2016), Hu et al. [63] (2012), and Nordmann et al. [18] (2006) in terms of the benefit on weight loss and lipids. These studies showed a similar benefit of low-carbohydrate diets on weight loss, HDL, and triglycerides, taken in consideration with their disadvantageous effects of increasing LDL and total cholesterol levels. Nevertheless, Mansoor et al. and Hu et al. did not stratify the outcomes by follow-up time and Nordmann et al. only stratified for after 6 and 12 months of follow-up. Our stratification at 3, 6, 12 and >12 months allowed for a more nuanced understanding of the effect of these diets depending on the duration of diet. These meta-analyses also included fewer studies and participants with the greatest number being in Hu et al. with 23 articles and 2788 participants [63]. The present study included 38 articles and 6499 participants.

The low-carbohydrate diet has shown benefits such as increased insulin sensitivity and decreased serum insulin concentrations [50,64]. This is expected, as carbohydrates are the major stimulants for insulin secretion [65]; this benefit might be associated with increased satiety supported by findings that foods with higher insulin response are less satiating [66,67,68]. Other benefits of a low-carbohydrate diet include a decrease in ghrelin and leptin and increased energy expenditure during weight loss, as found by Ebbeling et al. [69]. It is also important to consider other mechanisms such as energy expenditure, hormone release, adipogenesis, and fatty acid metabolism [42], which can result in the differences observed between the low-carbohydrate and low-fat diets.

Lipid levels of LDL, HDL, and TGs are used clinically as prognostic markers of CVD [70]. Decreasing fat intake is associated with increased carbohydrates, which can cause carbohydrate-induced hypertriglyceridemia [71,72]. This should be considered as a negative consequence of implementing a low-fat diet. The beneficial effects of increased HDL and decreased TGs are debated due to a lack of effectiveness of HDL cholesterol increase on treatment [70,73]. Moreover, the increase in LDL in the low-carbohydrate group replicated in previous meta-analyses is associated with an increase in saturated fat intake in low-carbohydrate diets [74,75]; saturated fatty acids are known as the dietary factor with the strongest impact on LDL cholesterol levels [70]. LDL is one of the most important biomarkers for CVD risk prediction and the target of the pharmaceutical strategies we use including statins and PCSK9 inhibitors [76,77,78]. Given the strong association of increased LDL with CVD, physicians should consider a patients’ lipid panels when recommending low-carbohydrate diets and inform the patient of potential consequences.

In line with previous meta-analyses [18,62,79], our analysis observed diminished differences between low-carbohydrate and low-fat diets at 24 months and the WMD decreased from −1.47 kg at 3–6 months to 0.83 kg at >12 months for weight loss. Poor adherence to the prescribed macronutrient composition in these diets might have contributed to diminished results beyond 12 months as non-adherence increases with an increased duration of time as suggested by Arora et al. [71]. Thus, it can be concluded that the low-carbohydrate diet is at least as effective as the low-fat diet up to 6 months and better between 6–12 months for weight loss. Non-adherence and physiological changes may be significant contributors to the decreasing effectiveness of the diet over time.

### 4.1. Quality of Evidence Used

Quality of the evidence used in this meta-analysis varied between the studies; most studies had “some concerns” (22/38). There were eight studies classified to be of “low risk” and eight studies as “high risk”. Analysing by category, most “some concerns” classifications were due to the randomisation process (24/38), deviations from intended interventions (24/38), and selection of reported results (22/38). This was partly due to studies not disclosing whether allocation sequences were concealed before assignment to intervention, in addition to the lack of blinding given the requirements of lifestyle interventions. Most “high” classifications were due to missing outcome data (5/38).

Moreover, only two studies examined outcomes beyond 12 months [36,39] and these had high attrition rates as retaining participants is difficult in long nutritional studies; both studies were classified to have a high risk of bias. 

### 4.2. Strengths and Limitations

Limitations of this meta-analysis included a search limited to English language publications, not including a search of grey literature, which may have caused trials to be missed. Additionally, as mentioned, only two studies analysed effects beyond 12 months [36,39], resulting in underpowered results for this time category. Moreover, the majority of the trials (32 out of 38) did not provide food for the duration of the trial, which lowered the adherence to chosen diet protocols. Some studies also did not provide any information on whether the participants engaged in any physical activity. Further subgroup analyses based on gender and ethnicity would have been provided beneficial insights into these diets. However, most studies did not stratify their outcomes by these categories meaning we were unable to perform such analyses. Limitations in our analysis included the fact that we did not take into account the correlation between time categories. Although cross-over trials include two periods of treatment compared to the parallel RCTs, all three crossover trials only provided overall results for the low-carb and low-fat arm; hence, these measures were used in our analysis.

Further limitations included that the diets did not account for the quality of the food consumed. A low-carbohydrate diet may make it easier to remove processed foods, which have refined carbohydrates such as white bread and pasta, as well as sugary drinks. Intake of ultra-processed foods has been linked with a higher risk of overall cardiovascular disease [80] as well as obesity [81,82,83]. It would be interesting to observe whether these results would be repeated for unprocessed, complex carbohydrate intake in the low-fat groups. 

This study also had several strengths. A strict protocol was followed in performing the meta-analysis: two reviewers independently reviewed articles and extracted data were verified by a second reviewer. All studies included were randomised controlled trials, which were subject to fewer biases in comparison to observational studies. Moreover, this meta-analysis had a sample size of 6499, which allowed for greater power in detecting statistically significant mean differences in our outcomes. The large number of included studies allowed us to assess publication bias. Strict definitions of low-carbohydrate diets (≤40% carbohydrate) and low-fat diets (≤30%) were used to prevent bias from subjective dietary classification. 

## 5. Conclusions

In conclusion, our meta-analysis found higher levels of weight loss for up to 1 year in participants on a low-carbohydrate diet compared to those on a low-fat diet, with an improved HDL profile, and improved TG profile; yet, less favourable changes in LDL and total cholesterol levels. The benefits of the low-carbohydrate diet on weight loss in the short-term must be balanced with potential consequences of raised LDL and total cholesterol in the long-term. None of the studies examined long-term clinical end-points such as cardiovascular disease or mortality. Dietary interventions and nutritional care are important as they integrate the social, physical, and mental well-being of the patient. Further emphasis should be placed on conducting long-term (>12 months) high-quality dietary RCTs, while providing information on food intake, calories, macronutrients, as well as physical activity to allow for better characterisation of the interaction of nutrition with obesity and cardiovascular health.

## Figures and Tables

**Table 1:** Study characteristics of randomised trials of low-fat vs. low-carbohydrate dietary interventions.

First Author	Randomised Participants	Country	Low-Fat Intervention	Low-Carb Intervention	Follow-Up Duration (Months)
Antonio De Luis, 2015 [29]	331	Spain	Standard protein hypocaloric: 55% carbohydrates, 27% fat, 20% protein	High Protein hypocaloric: 33% carbohydrate, 33% fat, 34% protein	9
Bazzano, 2014 [30]	148	USA	National Cholesterol Education Program Guidelines (<30% fat, 55% carbohydrates)	Low-carbohydrate diet	12
Bradley, 2009 [31]	27	UK	20% fat, 60% carbohydrate; ~500 kcal calorie deficit	60% fat, 20% carbohydrate; ~500 kcal calorie deficit	1.84
Brehm, 2003 [32]	53	USA	Energy-restricted, moderately low-fat diet with 55% carbohydrate, 15% protein, and 30% fat.	Ad libitum low carb, <20g carbohydrate/day; increase to 40–60 g/day if still in ketosis.	6
Brehm, 2005 [33]	50	USA	Energy-restricted, moderately low-fat diet with 55% carbohydrate, 15% protein, and 30% fat.	Ad libitum low carb, <20 g carbohydrate/day; increase to 40–60 g/day if still in ketosis.	4
Brinkworth, 2009 [34]	118	Australia	30% fat; isocaloric	Energy restricted (6–7 megajoules) low-carbohydrate (4%, 35%, and 61% of energy as carbohydrate, protein, and fat)	12
Cornier, 2005 [35]	44	USA	60% CHO, 20% fat, and 20% protein (high carbohydrate/low fat); energy restricted 400 kcal deficit	40% carbohydrate 40% fat, and 20% protein (low carbohydrate/ high fat); energy restricted 400kcal deficit	3.68
Dale, 2009 [36]	200	New Zealand	High-monounsaturated fat diet; 25% protein, 21% monounsaturated fat, 40% carbohydrate (Nurse supported or Intensive supported)	55% carbohydrates, 15–20% protein and 25–30% fat; encouraged to consume low-glycaemic food (Nurse supported or Intensive supported)	24
Dansinger, 2005 [37]	160	USA	Ornish, vegetarian diet containing 10% of calories from fat.	Atkins diet group, <20 g/day with gradual increase to 50 g/day	12
Foraker, 2014 [38]	79	USA	60% carbohydrates, 20% fat, 20% protein	40% carbohydrates, 30% fat, 30% protein	18
Foster, 2003 [24]	63	USA	60% carbohydrate, 25% fat, and 15% protein	Atkins diet group, 20 g/day with gradual increase until stable	12
Foster, 2010 [39]	307	USA	55% carbohydrates, 30% fat, 15% protein	Atkins diet group, 20 g/day for first 12 weeks with gradual of 5 g/day per week after	24
Frisch, 2009 [40]	200	Germany	>55% carbohydrate, <30% fat, 15% protein	<40% carbohydrates, >35% fat, 25% protein	12
Gardner, 2007 [41]	311	USA	(1) LEARN The LEARN group was instructed to follow a prudent diet that included 55% to 60% energy from carbohydrate <10% saturated fat (2) Ornish <10% fat	Atkins, <20 g/d or less of carbohydrate for “induction” (usually 2–3 months) and 50 g/d or less of carbohydrate for the subsequent “ongoing weight loss” phase.	12
Gardner, 2016 [42]	31	USA	Reduced intake of fat to 20 g/day to achieve lowest level of fat during first 8 weeks; in the second stage slowly add fat in increments of 5 g/d and hold for 1–4 weeks before adding another 5 g/day; third stage to identify lowest level they could maintain long term.	Reduced intake of carbohydrates to 20g/day to achieve lowest level of carbohydrates during first 8 weeks; in the second stage slowly add carbohydrates in increments of 5 g/d and hold for 1–4 weeks before adding another 5 g/day; third stage to identify lowest level they could maintain long term.	6
Gardner, 2018 [43]	632	USA	Reduced intake of fat to 20 g/day during first 8 weeks; slowly add carbohydrates in increments of 5–15 g/d per week until they reached lowest level of intake they could maintain indefinitely	Reduced intake of carbohydrates to 20 g/day during first 8 weeks; slowly add carbohydrates in increments of 5–15 g/d per week until they reached lowest level of intake they could maintain indefinitely	12
Halyburton, 2007 [44]	121	Australia	46% carbohydrate, 30% fat, 245 protein	4% carbohydrate, 61% fat (20% saturated fat), 35% protein	1.84
Haufe, 2011 [60]	174	Germany	20% fat, 0.8 g protein/kg body weight, and the remaining energy content provided by carbohydrates in the reduced fat group	90 g carbohydrates, 0.8 g protein/kg body weight, and a minimum of 30% fat in the reduced carbohydrate group	6
Jenkins, 2014 [11]	50	Canada USA	High-carbohydrate lacto-ovo vegetarian diet 58% carbohydrate, 25% fat, 16% protein	Low-carbohydrate vegan diet with 265% carbohydrates, 43% fat, 31% vegetable proteins	6
Keogh, 2007 [44]	44	Australia	60% carbohydrate, 20% fat, 20% protein	33% carbohydrate, 27% fat, 40% protein	3
Kirk, 2009 [26]	22	USA	≥180 g carbohydrates, 20% fat, 15% protein	≤50 g carbohydrates/day, 10% carbohydrates, 75% fat, 15% protein	2.75
McAuley, 2005 [45]	96	New Zealand	High-carbohydrate, high-fibre diet (control group) based on that recommended by Diabetes and Nutrition Study Group (DNSG) of the European Association for the Study of Diabetes (EASD) and the diet was implemented using the national healthy eating guidelines, with slight modifications	Atkins, in first 2 weeks <20 g/day of carbohydrates; during weeks 3 to 8 of the weight loss phase, carbohydrate was reintroduced by the addition of 5 g/day each week, so that a maximum of 50 g of carbohydrate per day was consumed by week 8.	3.68
McLaughlin, 2006 [46]	65	USA	16 week calorie restriction 60% carbohydrates, 25% fat, 15% protein Then 2 week weight maintenance with eucaloric diet based on weight and macronutrient content similar to hypocaloric diet	16 week calorie restriction 40% carbohydrates, 45% fat, 15% protein Then 2 week weight maintenance with eucaloric diet based on weight and macronutrient content similar to hypocaloric diet	4.14
Meckling, 2004 [47]	40	Canada	Low fat diet, eliminated high-fat dairy products and substitute with no-fat or low fat alternatives.	The goal of the low carbohydrate diet was to restrict carbohydrates to 50–70 g/d by gradually restricting carbohydrate intake from 100 g on d 0 to 50–70 g by d 5.	2.3
Nickols-Richardson, 2005 [48]	28	USA	60% carbohydrate, 25% fat, 15% protein	Atkins Nutritional Approach: during the first 2 weeks, consumed <20 g carbohydrate/day; thereafter, they increased their carbohydrate intake by 5 g/week to 40 g carbohydrate/day at week 6.	1.38
Phillips, 2008 [49]	28	USA	30% fat modelled after an American Heart Association diet	Atkins-style diet with 20 g/day carbohydrates supplemented with protein and fat content according to the Atkins’ diet recommendations	1.38
Ruth, 2013 [27]	55	USA	60% complex carbohydrates, 25% fat, 15% protein	≤40 g/day carbohydrates, 60% fat, 15% protein	12
Sacks, 2009 [7]	811	USA	Low Fat High Protein 55% carbohydrate, 20% fat, 25% protein Low Fat Average Protein 65% carbohydrate, 205 fat, 15% protein	High fat high protein 35% carbohydrate, 20% fat, 15% protein (High fat average protein group did not meet criteria)	24
Samaha, 2003 [50]	132	USA	Received instruction in accordance with the obesity-management guidelines of the National Heart, Lung, and Blood Institute, including caloric restriction to create a deficit of 500 calories/day, with ≤30% of total calories derived from fat.	≤30 g/day carbohydrates, no instruction on reducing total fat intake	6
Sharman, 2004 [51] *	15	USA	~55% carbohydrate, 25% fat, 20% protein	10% carbohydrates, 60% fat, 30% protein	1.5
Soenen, 2012 [52]	139	The Netherlands	High protein normal carbohydrate 50% carbohydrate, 30% fat, 20% protein (Additionally had a normal protein normal carbohydrate group that did not match selection criteria)	(1) High protein low carbohydrate 25% carbohydrate, 55% fat, 20% protein (2) Normal protein low carbohydrate 25% carbohydrate, 65% fat, 10% protein	12
Stern, 2004 [53]	132	USA	Reduced caloric intake by 500 calories per day, with less than 30% of calories derived from fat	<30 g/day carbohydrate	12
Varady, 2011 [54]	20	USA	55% carbohydrate, 25% fat, 20% protein	5% carbohydrate, 60% fat, 35% protein	1.5
Veum, 2017 [55]	46	Norway	53% carbohydrate, 30% fat, 17% protein	10% carbohydrate, 73% fat, 17% protein	3
Volek, 2003 [56] *	10	USA	Subjects consumed each diet for 4 weeks followed by a 4-week break before crossing over to the other diet. 55% carbohydrate, 25% protein, 20% fat	Subjects consumed each diet for 4 weeks followed by a 4-week break before crossing over to the other diet. 10% carbohydrate, 60% fat, 30% protein	1
Volek, 2004 [57] *	13	USA	55% carbohydrate, 25% protein, 20% fat	10% carbohydrate, 60% fat, 30% protein	1
Wal, 2007 [58]	125	USA	Moderate carbohydrate group	The Low carbohydrate group	3
Wood, 2012 [59]	42	USA	<30% fat with <10% saturated fat and <300mg/day dietary cholesterol	<50 g of carbohydrate per day, with no specific restrictions provided with respect to total or saturated fat consumption or dietary cholesterol consumption.	3

* Crossover studies.

**Table 2:** Age, BMI, presence of calorie restriction, food provision, and exercise for all of the included studies.

First Author, Year	Age (Years) Low-Carb	Age (Years) Low-Fat	BMI (kg/m^2^) Low-Carb	BMI (kg/m^2^) Low-Fat	Calorie Restriction (Y/N)	Food Provision (Y/N)	Physical Activity Prescribed (Y/N)
Antonio De Luis, 2015 [29]	50.5	49.9	35.4	35.1	Y	N	Y
Bazzano, 2014 [30]	45.8	47.8	35.2	35.6	N	N	N
Brehm, 2003 [32]	44.2	43.1	33.17	34.04	Y (low-fat only)	N	N
Brehm, 2005 [33]	44.8	41.4	32.8	33.5	Y (low-fat only)	N	N
Brinkworth, 2009 [34]	51.5	51.4	33.6	33.3	Y	Y	N
Cornier, 2005 [35] ^§^	41.3	43.5	33.1	30.8	Y	Y	N
Cornier, 2005 [35] ^§^	43.6	36.8	32.2	33	Y	Y	N
Dale, 2009 [36]	45	45	31.9	31.8	N	N	Y
Dansinger, 2005 [37]	47	49	35	35	N	N	Y
Foraker, 2014 [38]	41.9	40.9	30.1	30.5	Y	N	Y
Foster, 2003 [24]	44	44.2	33.9	34.4	Y (low-fat only)	N	N
Foster, 2010 [39]	46.2	44.9	36.1	36.1	Y (low-fat only)	N	Y
Frisch, 2009 [40]	47	47	33.5	33.8	Y	N	N
Gardner, 2007 [41] ^||^	42	40	32	31	N	N	Y
Gardner, 2007 [41] ^||^	42	42	32	32	N	N	Y
Gardner, 2016 [42] ^†^	42	44	34.2	35	N	N	Y
Gardner, 2016 [42] ^†^	43	41	31.2	32.6	N	N	Y
Gardner, 2018 [43]	40.2	39.3	33.3	33.4	N	N	Y
Halyburton, 2007 [25]	50.6	49.8	33.3	33.8	Y	Y	N
Haufe, 2011 [60]	NA	NA	NA	NA	Y	N	N
Jenkins, 2014 [11]	57.6	55.3	31.1	31.1	Y	N	N
Keogh, 2007 [44]	50·1	46.9	32·6	33.2	Y	N	N
Kirk, 2009 [26]	41.8	45.4	36.1	36.9	Y	N	N
McAuley, 2005 [45]	45	45	36	36.6	N	N	N
McLaughlin, 2006 [46]	48	53	32.3	33	Y	N	N
Meckling, 2004 [47]	41.2	43.2	32.2	32.2	Y (low-fat only)	N	N
Nickols-Richardson, 2005 [48]	38.8	40.1	31.1	**30.3**	Y (low-fat only)	N	N
Phillips, 2008 [49]	33	38	34	33.8	Y	N	N
Ruth, 2013 [27]	43.5	41.5	37.1	35.9	Y	N	N
Sacks, 2009 [7] ^#^	51	50	33	33	Y	N	Y
Sacks, 2009 [7] ^#^	51	51	33	33	Y	N	Y
Samaha, 2003 [50]	53	54	42.9	42.9	Y (low-carb only)	N	N
Sharman, 2004 [51]	33.2	33.2	34.3	34.3	Y	N	N
Soenen, 2012 [52] ^¶^	NA	NA	36.6	37.5	N	N	N
Soenen, 2012 [52] ^¶^	NA	NA	37	37.5	N	N	N
Stern, 2004 [53]	55	55	43.6	42.3	Y (low-fat only)	N	N
Varady, 2011 [54]	35	36	33	34	Y	Y	N
Veum, 2017 [55] *	40.3	40.2	34.1	33.6	N*	N	N
Volek, 2003 [56]	26.3	26.3	22	22	N	N	N
Volek, 2004 [57]	34	34	29.6	29.6	Y	N	N
Wal, 2007 [58]	50.5	g49.6	33.1	37.3	Y	Y	N
Wood, 2012 [59] ^‡^	58.6	58.4	34	35.2	N (low-fat only)	N	Y ^‡^

**^§^ Cornier 2005** Divided into insulin sensitive and insulin resistant groups listed, respectively. **^||^ Gardner 2007** Three diet groups met our inclusion criteria; two low-fat groups were compared against one low-carb group. **^†^ Gardner 2016** Subdivided into insulin resistant and insulin sensitive groups listed, respectively. Participants on the low-carbohydrate diet were asked to consume one avocado per day which was provided by the Hass Avocado board. ^#^ **Sacks 2009** Three diet groups met our inclusion criteria; two low-fat groups were compared against one low-carb group. ^¶^ **Soenen 2012** Three diet groups met our inclusion criteria, two low-carbohydrate groups were compared against one low-fat group. * Veum 2017: aimed to study macronutrient difference, not energy restriction; participants asked to consume 8750 kJ/day (~2090 kcal/day). ^‡^ **Wood 2012** Participants were stratified into a physical activity and non-physical activity group.

## Supplementary Materials

Supplementary files 1–3 are available online at https://www.mdpi.com/2072-6643/12/12/3774/s1, Supplementary File 1: Search Terms; Supplementary File 2: Risk of Bias; Supplementary File 3: Funnel Plots for Publication Bias.
