# Supplementary material for: The Effect of Low-Fat and Low-Carbohydrate Diets on Weight Loss and Lipid Levels: A Systematic Review and Meta-Analysis
Source: Nutrients. 2020 Dec 9;12(12):3774. doi: 10.3390/nu12123774 (PMC7763365; doi:10.3390/nu12123774)
Supplement: Supplementary file 1 [file nutrients-12-03774-s001.zip › Supplementary Files/Supplemental File 1.docx]

**Supplemental File 1: Search Terms**

**MEDLINE**

(((((((((Diet, Carbohydrate-Restricted*[MeSH Terms]) OR Diet, Fat-Restricted*[MeSH Terms]) OR diet, low carbohydrate[MeSH Terms]) OR low carbohydrate diet[MeSH Terms]) OR diet, low fat[MeSH Terms]) OR low fat diet[MeSH Terms]) OR low carbohydrate[Title/Abstract]) OR low fat[Title/Abstract]) OR carbohydrate restructed[Title/Abstract]) OR fat restricted[Title/Abstract] AND

 ((((((cardiovascular outcomes) OR cardiovascular risk factors) OR weight loss*[MeSH Terms]) OR body weight[MeSH Terms]) OR Obesity/diet therapy*[MeSH Terms]) OR treatment outcome[MeSH Terms]) OR cardiovascular outcome[Title/Abstract] Sort by: Best Match

* Restricted to randomised controlled trials
