# Supplementary material for: The Effect of Low-Fat and Low-Carbohydrate Diets on Weight Loss and Lipid Levels: A Systematic Review and Meta-Analysis
Source: Nutrients. 2020 Dec 9;12(12):3774. doi: 10.3390/nu12123774 (PMC7763365; doi:10.3390/nu12123774)
Supplement: Supplementary file 1 [file nutrients-12-03774-s001.zip › Supplementary Files/Supplemental File 2.docx]

**Supplemental File 2: Risk of Bias Table**

| Reference | Randomization process | Deviations from intended interventions | Missing outcome data | Measurement of the outcome | Selection of the reported result | Overall Bias |
| --- | --- | --- | --- | --- | --- | --- |
| Bazzano2014 | Low | Some concerns | Some concerns | Some concerns | Some concerns | Some concerns |
| Bradley2009 | Some concerns | Some concerns | Low | Some concerns | Some concerns | Some concerns |
| Brehm2003 | Low | Some concerns | Low | Some concerns | Some concerns | Some concerns |
| Brehm2005 | Some concerns | Some concerns | Low | Some concerns | Some concerns | Some concerns |
| Brinkworth2009 | Some concerns | Some concerns | Low | Low | Low | Some concerns |
| Cornier2005 | Some concerns | Low | Low | Low | Some concerns | Some concerns |
| Dale2009 | Low | Low | Low | Low | Some concerns | Some concerns |
| Dansinger2005 | Low | Low | High | Some concerns | Some concerns | High |
| DeLuis2015 | Some concerns | Low | Low | Low | Some concerns | Some concerns |
| Foraker2014 | Some concerns | Some concerns | Low | Some concerns | Some concerns | Some concerns |
| Foster2003 | Some concerns | Some concerns | Low | Some concerns | Some concerns | Some concerns |
| Foster2010 | High | Some concerns | High | Low | Some concerns | High |
| Frisch2009 | Some concerns | Some concerns | High | Low | Some concerns | Some concerns |
| Gardner2007 | Low | Low | High | Low | Some concerns | High |
| Gardner2016 | Some concerns | High | Low | Some concerns | High | High |
| Gardner2018 | Low | Some concerns | Low | Low | Low | Some concerns |
| Halyburton2007 | Some concerns | Some concerns | Low | Low | Some concerns | Some concerns |
| Haufe 2011 | Low | Low | Low | Low | Low | Low |
| Jenkins2014 | Some concerns | Low | Low | Some concerns | Some concerns | Some concerns |
| Keogh2007 | Some concerns | Some concerns | Low | Low | Some concerns | Some concerns |
| Kirk2009 | Some concerns | Some concerns | Low | Low | Some concerns | Some concerns |
| McAuley 2005 | Low | Some concerns | Low | Low | Some concerns | Some concerns |
| McLaughlin 2006 | Some concerns | Some concerns | Some concerns | Low | Low | High |
| Meckling 2004 | Low | High | Low | Low | Some concerns | High |
| Nickols-Richardson 2005 | Some concerns | Some concerns | Low | Low | Low | Low |
| Phillips 2008 | Low | Low | Low | Low | Low | Low |
| Ruth MR 2013 | Some concerns | Some concerns | Low | Low | Low | Low |
| Sacks FM 2009 | Low | Low | Low | Low | Low | Low |
| Samaha 2003 | Low | Some concerns | Low | Low | Low | Low |
| Sharman MJ 2004 | Some concerns | Some concerns | Low | Low | Some concerns | Some concerns |
| Soenen 2012 | Some concerns | Low | Low | Low | Low | Low |
| Stern 2004 | Some concerns | Some concerns | Low | Low | Low | Some concerns |
| Varady 2011 | Low | Low | Low | Low | Low | Low |
| Veum 2017 | Some concerns | Some concerns | Low | Low | High | High |
| Volek 2003 | Some concerns | Some concerns | Low | Low | Some concerns | Some concerns |
| Volek 2004 | Some concerns | Some concerns | Low | Low | Some concerns | Some concerns |
| Wal JS 2007 | Some concerns | Some concerns | Low | Low | Low | Some concerns |
| Wood 2012 | Some concerns | High | Some concerns | Low | Low | High |
