# Supplementary material for: The Effect of Low-Fat and Low-Carbohydrate Diets on Weight Loss and Lipid Levels: A Systematic Review and Meta-Analysis
Source: Nutrients. 2020 Dec 9;12(12):3774. doi: 10.3390/nu12123774 (PMC7763365; doi:10.3390/nu12123774)

**Supplemental File 3: Funnel Plots for Publication Bias**

**Weight**


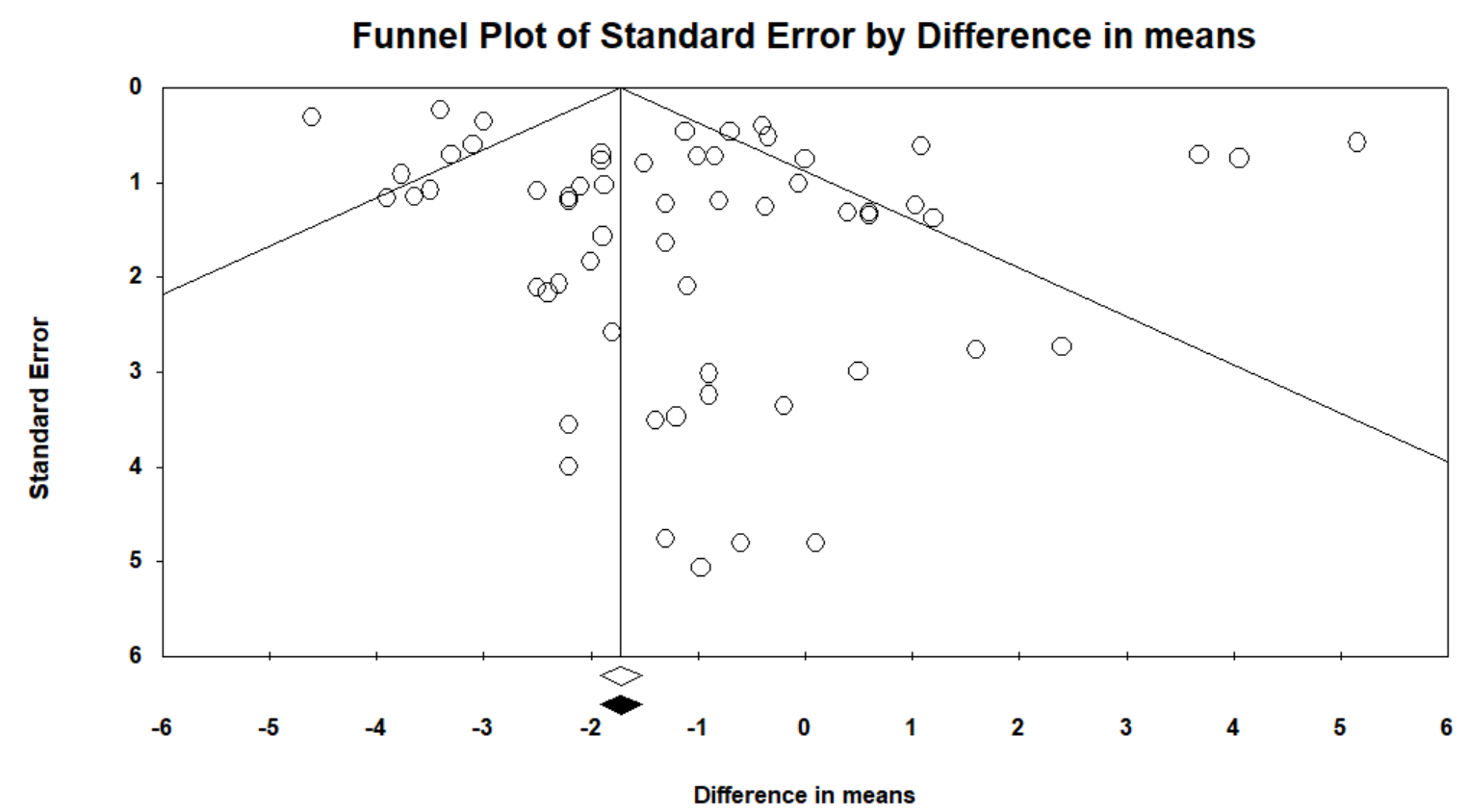


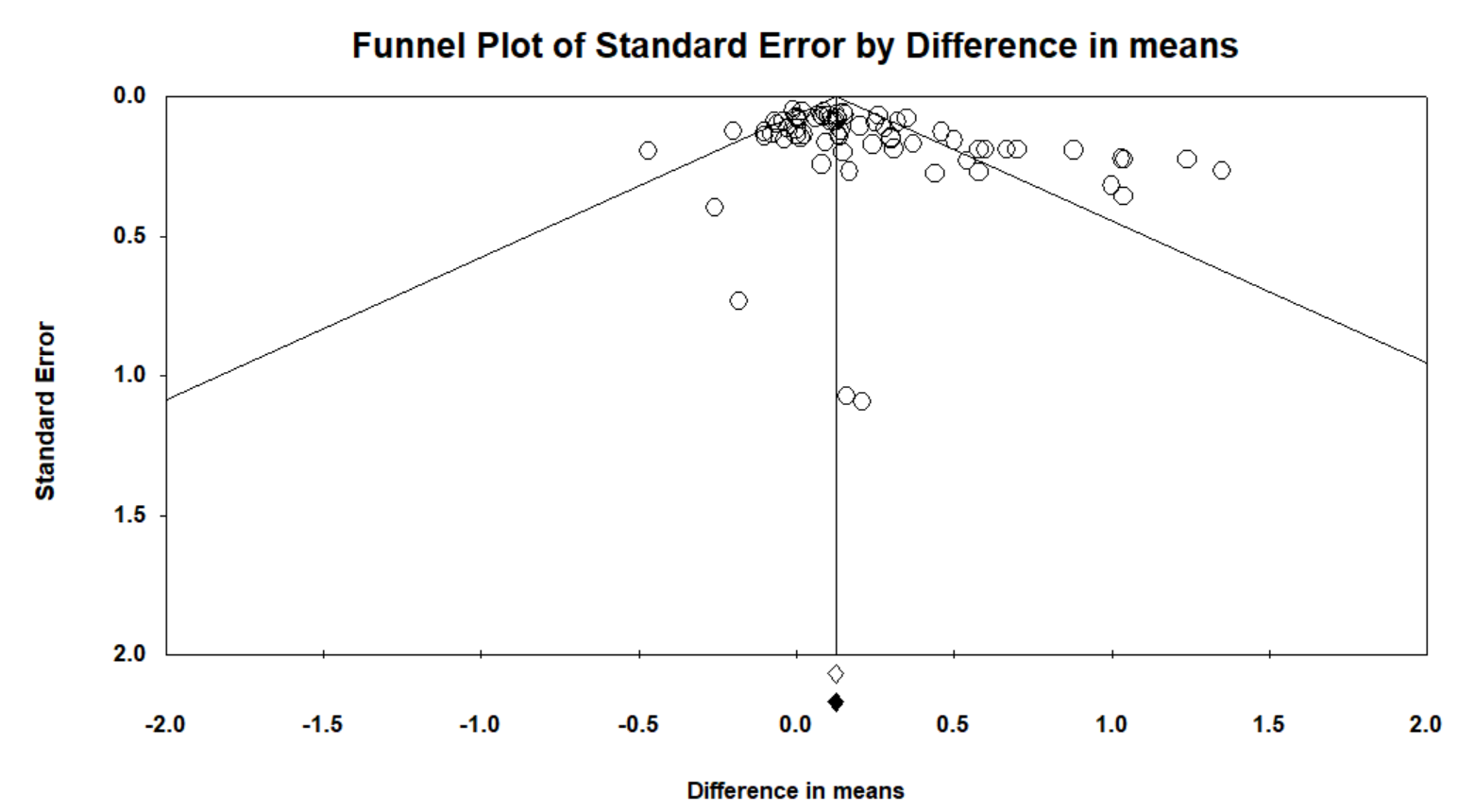
**LDL**


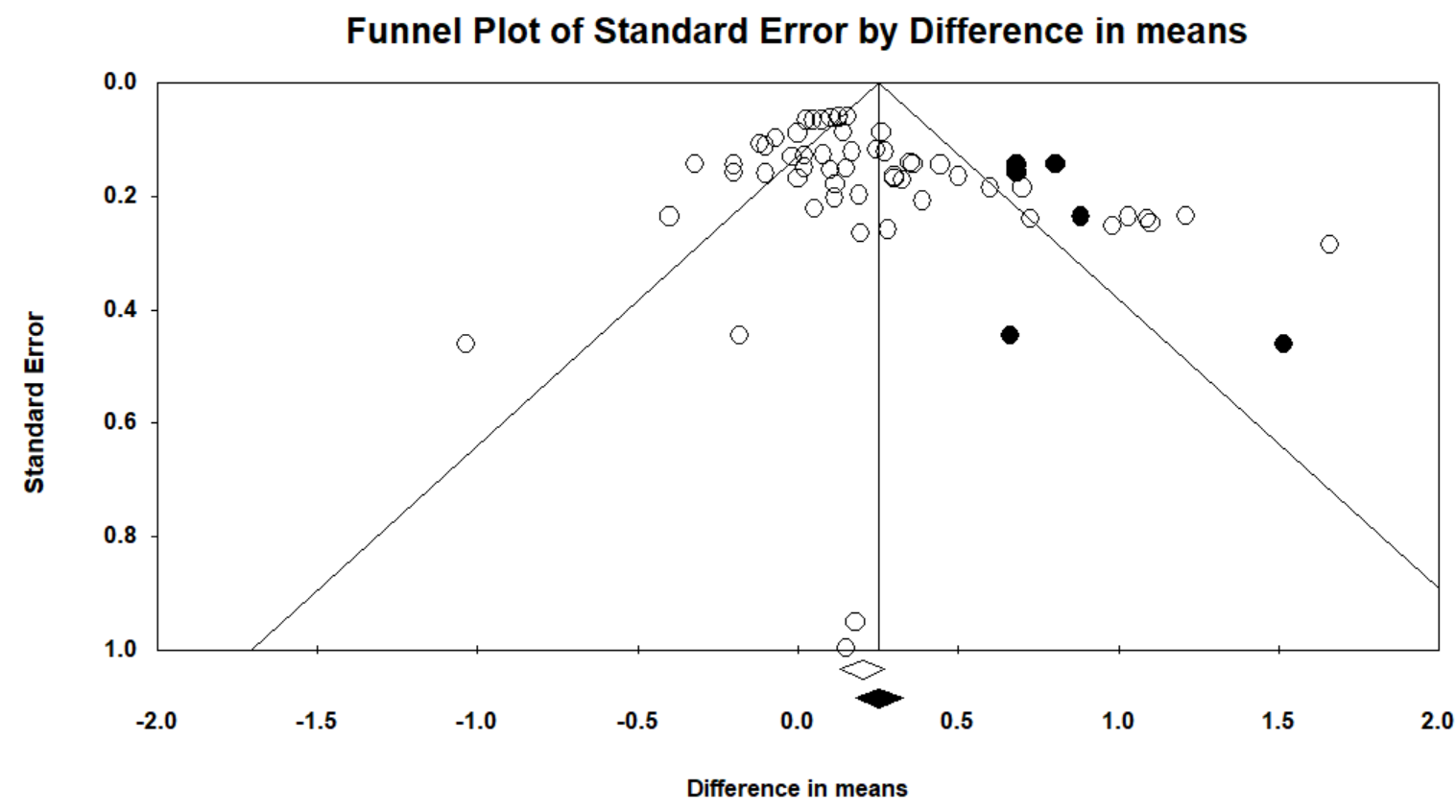
**Total Cholesterol**

**HDL**


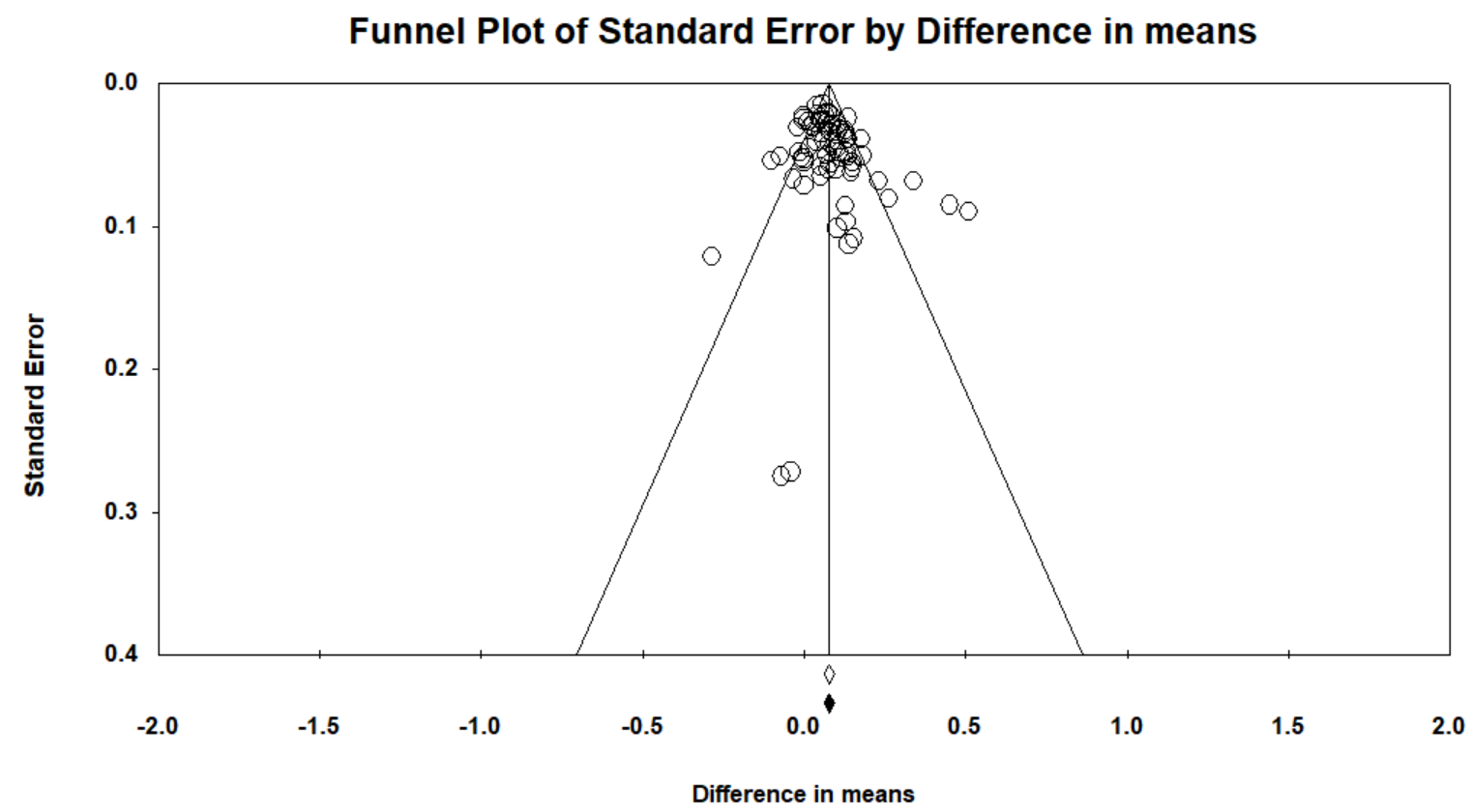


**Triglycerides**


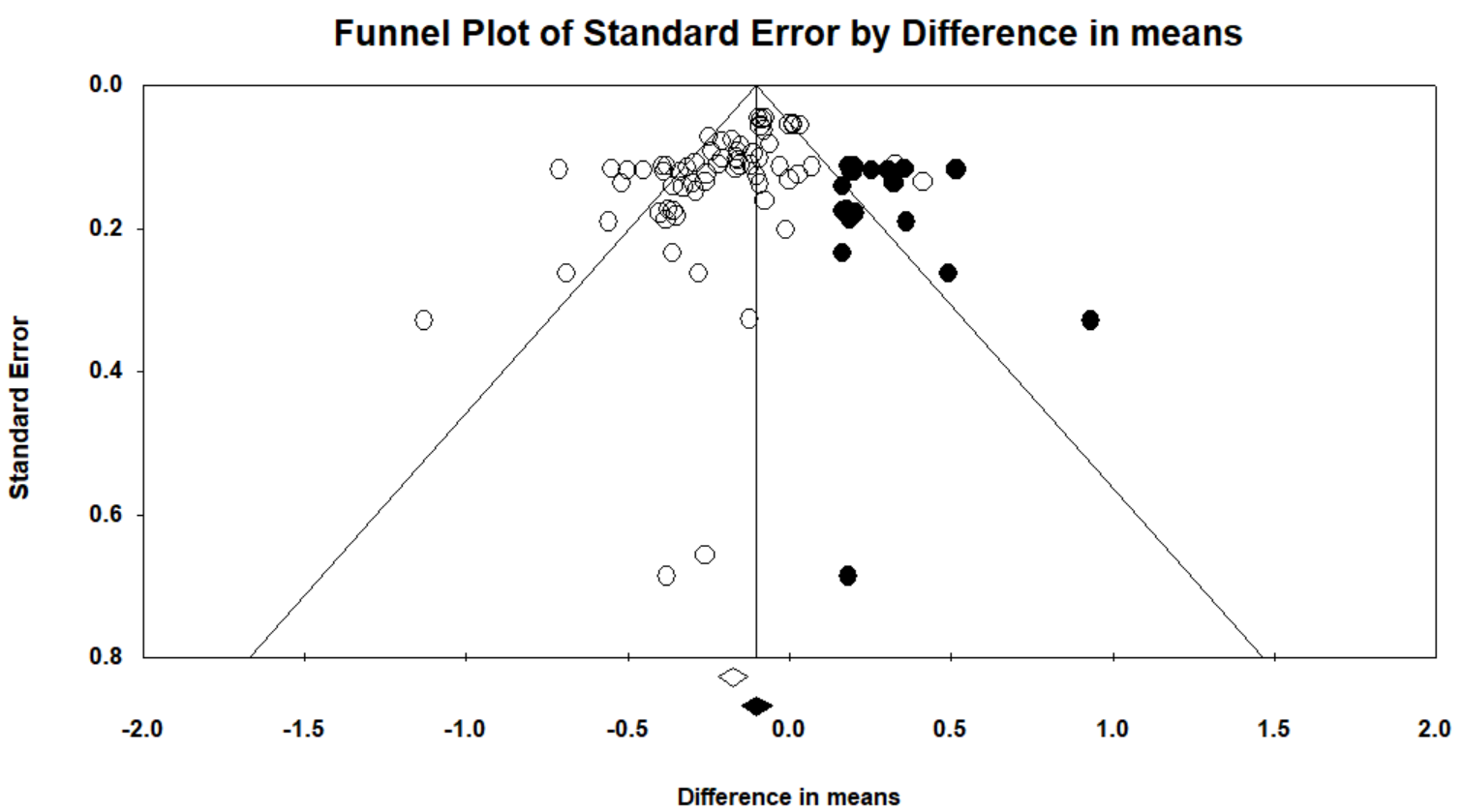

Supplement: Supplementary file 1 [file nutrients-12-03774-s001.zip › Supplementary Files/Supplemental File 3.docx]
